# Co-occurrence of *Babesia microti*, *Bartonella *spp., *Borrelia burgdorferi *s.l. and *Anaplasma phagocytophilum *in rodents from Lower Silesia, Poland

**DOI:** 10.1186/1756-3305-7-S1-O4

**Published:** 2014-04-01

**Authors:** J Hildebrand, K Buńkowska-Gawlik, A Perec-Matysiak

**Affiliations:** 1 Department of Parasitology, University of Wrocław; Przybyszewskiego Str. 63, 51-148 Wrocław, Poland

## 

This study aims to establish the relative contribution of rodent populations from diverse habitats to the occurrence of rodent-borne pathogens of public health significance. Rodents (n = 492) represented by *Apodemus agrarius*, *A. flavicollis *and *Myodes glareolus*, were captured in live traps in four localities of south-western Poland (2009-2012). For the analysis of co-occurrence of pathogens, *Babesia microti, Borrelia burgdorferi *s.l., *Bartonella *spp. and *Anaplasma phagocytophilum*, both blood and spleen samples were obtained from selected rodent specimens. The choice of genetic markers and primers was based on the literature data and our preliminary results. Conventional PCR was used for the detection of DNA of examined pathogens. Selected PCR positive products were purified and sequenced. BLAST searches were conducted in order to elucidate any homologies with previously deposited sequences in GenBank.

The DNA of pathogens was detected in 66.7% of the rodents tested. We observed that among infected rodents, 40.5% were infected with at least two pathogens, while only 4.7% with all four pathogens. All three of the rodent species were infected with each of the examined pathogens. In examined rodent populations the prevalence of *B. microti *was 40.0%, *Bartonella *spp. 37.7%, *B. burgdorferi *s.l. 28.2% and *A. phagocytophilum *17.7%.

In each of the tissue samples (blood or spleen), the prevalences of *Babesia microti *and *Bartonella *spp. were recorded as comparable, on rather high levels. Interestingly, the occurrence of these pathogens in both blood and spleen was only detected in 13% of the rodents.

While examining the blood and spleen-derived DNA, it was found that as many as 92.7% of *A. agrarius *harbored at least one pathogen. Co-occurrence of 3-4 pathogens was most common in this rodent species (32.1% infected).

By examining the spleen and blood samples of rodents at the same time, we estimate that the prevalence of pathogens in these rodents is higher than the literature indicates. This would implicate the examined rodent species as a significant reservoir of pathogens with zoonotic potential. Additionally, the role of *A. agrarius*, now widespread in some regions of Europe, as a reservoir host needs to be emphasized. *A. agrarius *can act as a bridge between woodland habitats and periurban environments frequented by humans.

